# Aging oocytes: exploring apoptosis and its impact on embryonic development in common carp (*Cyprinus carpio*)

**DOI:** 10.1093/jas/skaf002

**Published:** 2025-01-06

**Authors:** Essaikiammal Sodalai Muthu Konar, Sebastian Brachs, Knut Mai, Swapnil Gorakh Waghmare, Tomas Policar, Azadeh Mohagheghi Samarin, Azin Mohagheghi Samarin

**Affiliations:** Research Institute of Fish Culture and Hydrobiology, South Bohemian Research Center of Aquaculture and Biodiversity of Hydrocenoses, Faculty of Fisheries and Protection of Waters, University of South Bohemia in Ceske Budejovice, 389 01 Vodňany, Czech Republic; Department of Endocrinology and Metabolism, Charité–Universitätsmedizin Berlin, Corporate Member of Freie Universität Berlin and Humboldt-Universität zu Berlin, 10115 Berlin, Germany; DZHK (German Centre for Cardiovascular Research), Partner Site Berlin, 10115 Berlin, Germany; Department of Endocrinology and Metabolism, Charité–Universitätsmedizin Berlin, Corporate Member of Freie Universität Berlin and Humboldt-Universität zu Berlin, 10115 Berlin, Germany; DZHK (German Centre for Cardiovascular Research), Partner Site Berlin, 10115 Berlin, Germany; Research Institute of Fish Culture and Hydrobiology, South Bohemian Research Center of Aquaculture and Biodiversity of Hydrocenoses, Faculty of Fisheries and Protection of Waters, University of South Bohemia in Ceske Budejovice, 389 01 Vodňany, Czech Republic; Research Institute of Fish Culture and Hydrobiology, South Bohemian Research Center of Aquaculture and Biodiversity of Hydrocenoses, Faculty of Fisheries and Protection of Waters, University of South Bohemia in Ceske Budejovice, 389 01 Vodňany, Czech Republic; Research Institute of Fish Culture and Hydrobiology, South Bohemian Research Center of Aquaculture and Biodiversity of Hydrocenoses, Faculty of Fisheries and Protection of Waters, University of South Bohemia in Ceske Budejovice, 389 01 Vodňany, Czech Republic; Research Institute of Fish Culture and Hydrobiology, South Bohemian Research Center of Aquaculture and Biodiversity of Hydrocenoses, Faculty of Fisheries and Protection of Waters, University of South Bohemia in Ceske Budejovice, 389 01 Vodňany, Czech Republic

**Keywords:** apoptosis, caspases, early blastula embryos, fish, in-vitro-aged oocytes, spontaneous activation

## Abstract

Ovulation, fertilization, and embryo development are orchestrated and synchronized processes essential for the optimal health of offspring. Postovulatory aging disrupts this synchronization and impairs oocyte quality. In addition, oocyte aging causes fertilization loss and poor embryo development. This investigation aimed to unravel the endpoint of in vitro oocyte aging in common carp (*Cyprinus carpio*) to understand the involvement of apoptosis in postovulatory oocyte death. It was observed that the fertilization ability significantly declined (*P* < 0.001) at 8-h poststripping (**HPS**), subsequently triggering apoptosis in the advanced stage of oocyte aging, i.e., 48 HPS. This process included an increase in proapoptotic transcripts (*fas*, *bax*, *cathepsin D*, *caspase 8*, *caspase 9*, and *caspase 3a*) (*P* < 0.05), elevated levels of caspase 3 protein (*P* < 0.05), and activation of caspase 3 enzyme (*P* < 0.001), a key player in apoptosis, in aging oocytes. Furthermore, the effects of oocyte aging on the embryonic apoptosis machinery were examined in embryos at 5-h postfertilization (**HPF**) and 24 HPF derived from fresh and aged oocytes. Expression of apoptotic genes and caspase enzyme activity remained at the basal level in 5 HPF (early blastula embryos) from both fresh and aged oocytes. In contrast, the zymogenic and active forms of caspase 3 increased in 24 HPF embryos from 8-h-aged oocytes (*P* < 0.01) compared with those from fresh oocytes. Thus, apoptosis intensified in 24 HPF embryos from aged oocytes without affecting the apoptotic machinery of early blastula embryos. Our findings demonstrate that apoptosis initiated by the Fas/FasL system is an important physiological process accompanying oocyte aging in common carp.

## Teaser

Oocyte aging causes loss of fertilization capacity and death by apoptosis in the advanced stage of aging. Oocyte aging also affects the apoptosis mechanism in embryo development, which could be the reason behind the high malformation rate.

## Introduction

Apoptosis is a critical process in the development of all multicellular organisms. It is a programmed, energy-dependent, coordinated, and endogenous mechanism essential for cell development and tissue homeostasis (Wyllie et al., 1980; Norbury and Hickson, 2001). Morphological changes during apoptosis include cell shrinkage, pyknosis, chromatin condensation, karyorrhexis, membrane blebbing, and the formation of apoptotic bodies (reviewed by Elmore, 2007). Distinctive biochemical characteristics of apoptosis are the activation of caspases, cleavage of intracellular substrates, DNA fragmentation, and externalization of phosphatidylserine (reviewed by Elmore, 2007). Although the initiation phase varies among cells, it ultimately leads to caspase activation and apoptotic body formation. The proteins and signaling molecules involved in apoptosis are evolutionarily conserved (Metzstein et al., 1998). The two well-known signaling mechanisms of apoptosis are the extrinsic and intrinsic pathways. The extrinsic pathway is triggered by the ligation of death receptors from the Tumor Necrosis Factor α family (Ashkenazi and Dixit, 1998). The intrinsic pathway is activated by cellular damage, decreased survival factors, and oxidative stress via mitochondrial protein release (Schultz and Harrington, 2003). Apoptotic pathways can also be categorized as caspase-dependent or caspase-independent (Zhao, 2012).

Apoptosis is involved in the elimination of atretic follicles during maturation and postovulatory follicles after ovulation, which is crucial for maintaining ovarian homeostasis (Tilly, 1996). While the role of apoptosis in regulating female reproduction has been extensively studied in *Drosophila melanogaster*, *Caenorhabditis elegans*, and *Mus musculus* (De Pol et al., 1998; Gumienny et al., 1999; Matova and Cooley, 2001), it remains unexplored in fish. Although the reproductive behaviors and strategies of fish differ significantly from those of amphibians, reptiles, and mammals, the fundamental processes underlying gametogenesis remain similar (Urbatzka et al., 2011). In fish, the journey of the egg starts from primordial germ cells to oogonia, develops into a primary oocyte, and matures into metaphase-arrested oocytes, which are ready for ovulation (Lubzens et al., 2010). After a distinct vitality window, depending on the species, ovulated oocytes undergo progressive quality deterioration, known as postovulatory aging. This aging process leads to changes in maternally incorporated components of the egg, resulting in critical phenotypic and functional changes in the progeny (Takahashi et al., 2011).

Previous studies on bovines, pigs, and mice have suggested that oxidative damage, mitochondrial dysfunction, poly(A) tail deadenylation of maternal genes, epigenetic changes, and apoptosis are involved in oocyte aging (reviewed by Di Nisio et al., 2022). While oxidative stress is not likely the main initiator of fish oocyte aging (Samarin et al., 2019a, 2019b), epigenetic modifications, such as posttranslational histone acetylation and DNA methylation, were altered in aged fish gametes (Cheng et al., 2021; Waghmare et al., 2021b; Samarin et al., 2023). Despite these studies, the molecular mechanisms accelerating fish oocyte aging after ovulation remain unclear. This study aims to investigate the involvement of apoptosis during oocyte aging. The fertilization capacity of oocytes declines with increased storage, but a small proportion can still be fertilized, albeit with compromised embryo quality (e.g., Samarin et al., 2015a, 2016; Waghmare et al., 2021a). Embryos developed from postovulatory aged oocytes exhibit abnormalities such as structural malformations, ploidy anomalies, reduced reproductive fitness, and shorter lifespans (Tarín et al., 2002; Takahashi et al., 2009; Samarin et al., 2016). The decline in embryo quality associated with aged oocytes is influenced by several factors, with mitochondrial dysfunction being one of the most critical contributors (Tarin et al., 2000). However, the precise mechanisms behind poor embryo development due to oocyte aging are not well understood. Apoptosis is the key mechanism in shaping tissues and eliminating superfluous cells during development in all vertebrates (Cole and Ross, 2001). It plays a major role in sex differentiation in many species, which develop ovary-like gonads irrespective of genotypic sex and show hermaphroditism in the juvenile stage (Uchida et al., 2002). Therefore, it is important to investigate the apoptotic machinery in embryos developed from postovulatory aged oocytes to understand its impact on embryonic development.

Fish is a promising model for studying oocyte aging due to their intrinsic nature of external fertilization, simple in vitro gamete manipulation, and high fecundity. According to the FAO, common carp (*Cyprinus carpio*) is one of the most commonly cultured freshwater fish species globally and is a popular model in OECD guidelines (FAO, 2009). Common carp gametes are widely used for biotechnological applications, specific breeding strategies, and conservation of genetic resources (Jin et al., 2021). In this study, the oocytes were stored in vitro at 20 °C for 0-, 4-, 8-, 12-, 28-, 48-, and 72-h poststripping (**HPS**) without any artificial storage medium. The effects of aging on egg development were examined, through fertilization, hatching, and malformation rates. To study the possible involvement of apoptosis in the deterioration of aged oocytes, we analyzed the differential expression of apoptosis-related genes and the zymogenic and active forms of caspases in in-vitro-aged oocytes, 5-h postfertilization (**HPF**) and 24 HPF embryos originating from fresh and aged oocytes.

## Materials and Methods

### Compliance with ethical standards

All methodological protocols, experimental manipulations, and sampling procedures used in the present study were approved by the expert committee of the Institutional Animal Care and Use Committee of the University of South Bohemia, Czech Republic. The coauthor Tomas Policar of this study deals with the manipulation and artificial reproduction of fish and holds a certificate (certificate no. CZ01660) giving the authorization to work with laboratory animals according to section 15d paragraph 3 of Act no. 246/1992 Coll. For the purposes of stripping gametes, fish were anesthetized with 0.05% tricaine methanesulfonate (MS‐222; Sigma‐Aldrich, Missouri, USA) to ensure their welfare and minimize any associated stress.

### Ethics approval

The experiment was carried out under controlled conditions of Recirculating Aquaculture System (**RAS**) in the Laboratory of Intensive Aquaculture, which is part of the University of South Bohemia, Faculty of Fisheries and Protection of Waters (Vodňany, Czech Republic). All fish manipulations during the experiment were governed by valid legislative regulations of the Czech Republic (Act No. 166/1996 and No. 246/1992); the permit was issued No. 58672/2020-MZE-18134 and No. 33446/2020-MZE-18134 in the NAZV QK22020144 project.

### Animal maintenance and gametes collection

The brood stock was prepared following the protocol of Samarin et al. (2015). Briefly, experimental fish were maintained in an RAS with a gradual increase in temperature from 16 to 20 °C. After a 3-day acclimation period, hormonal manipulation was performed using carp pituitary homogenate (**CPH**) (Klatovské rybářství a.s., Czech Republic) according to Horváth et al. (1985). Female brood fish received an intramuscular injection of CPH (0.3-mg/kg body weight) at 18 °C, followed by a second dose of CPH (3.5-mg/kg body weight) 12 h later at 20 °C. Male brood fish were injected with a single dose of CPH (4.5-mg/kg body weight) simultaneously with the second injection for females. After 10 h from the second dose, females were examined for ovulation every 2 h. Six females that ovulated within 2 h were used for the experiment.

### In vitro oocyte aging

**Figure 1.**
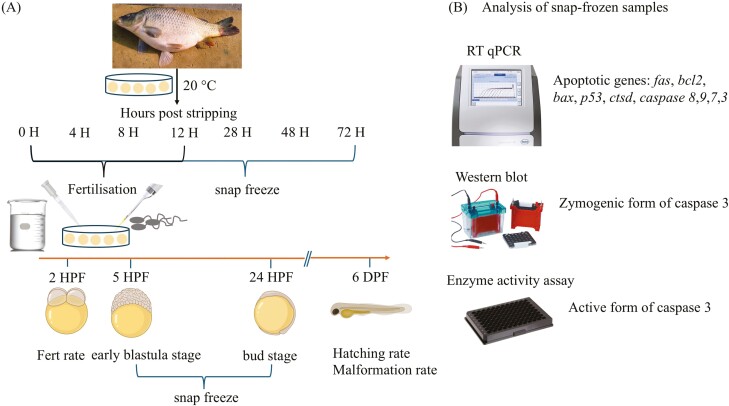
Schematic representation of experimental design to study the involvement of apoptosis in oocyte aging in common carp (*Cyprinus carpio*). (A) represents the sampling scheme and (B) represents the molecular and biochemical assays performed on the oocytes and embryo samples. Fert rate: Fertilization rate, DPF: Days Post Fertilization. Source icons from biorender.

The protocol for in vitro storage of oocytes was adapted from Samarin et al. (2019a). Egg aliquots from six females were stored two layers deep in ovarian fluid in sterile 6-well cell culture plates without artificial media or extender. The plates were covered with lids and placed in a humidified incubator at 20 °C. A humidified atmosphere was maintained by placing a few water-filled plates in the incubator. Oocyte samples were snap-frozen in liquid nitrogen at 0, 4, 8, 12, 28, 48, and 72 HPS and stored at −80 °C until further use (Figure 1).

### Artificial insemination and examination of egg developmental indices

Milt was collected from three fully mature males, pooled to compensate for individual paternal effects, and stored at 4 °C until use. A subportion of oocytes (2 g) from 0, 4, 8, and 12 HPS batches were artificially inseminated by adding 300-µL milt and 4,000-µL hatchery water following the protocol of Samarin et al. (2015). All batches of fertilized eggs were incubated separately in small, rectangular-shaped tanks (4.5-L capacity) with recirculating water at 20 ± 0.5 °C. Fertilization, hatching, and malformation rates were used as indices of egg viability (Lahnsteiner and Weismann, 1999; Goetz and Coffman, 2000) and calculated following Samarin et al. (2015). The fertilization rate was defined by the number of eggs showing cleavage to the total number of oocytes inseminated. As the apoptosis is inactive until the gastrulation stage (Ikegami et al., 1999; Negron and Lockshin, 2004), the embryo samples were collected at 5 HPF (early blastula) and 24 HPF (bud stage). The 5 HPF and 24 HPF embryos, which developed normally under Nikon SMZ745T stereomicroscope (Nikon, Japan), were washed with molecular-grade water, snap-frozen in liquid nitrogen, and stored at −80 °C (Figure 1). The hatching rate was determined by counting the number of hatched larvae 6 days after fertilization relative to the total number of inseminated eggs. Malformed larvae (e.g., spinal cord torsion, yolk sac, eye deformations) were identified using a stereomicroscope after hatching and counted to determine malformation rates.

### Gene expression analysis

#### RNA extraction and cDNA synthesis

Total RNA was isolated from 20 mg of oocytes and 20 embryos using Trizol (Thermo Fisher Scientific, USA) according to the manufacturer’s instructions. RNA concentration and quality were assessed using a Nanodrop 2000 (Thermo Fisher Scientific, USA). The isolated RNA was treated with DNase I (Amplification Grade, Thermo Fisher Scientific, USA), and 1,000 ng were reverse transcribed to cDNA using the Maxima First Strand cDNA Synthesis Kit (Thermo Fisher Scientific, USA) with 10-min incubation at 25 °C, followed by 15 min at 50 °C, and termination for 5 min at 85 °C.

#### Quantitative RT–PCR

Quantitative mRNA expression of apoptosis-related genes was analyzed using a LightCycler 96 (Roche, Germany). Primers were designed using Primer3, purchased from Eurofins Genomics (Germany), and validated by melt curve analyses (Table 1). Each reaction contained 2-µL diluted cDNA (1:10), 1 µL forward and reverse primers (500 nM), 2 µL nuclease-free water, and 5 µL PowerUp SYBR Green Master Mix (Thermo Fisher Scientific, USA). Data were collected from six replicates with three parallel repetitions. Relative gene expression was analyzed using the 2^-ΔΔCt^ method and normalized to gapdh (Livak and Schmittgen, 2001).

**Table 1.** primer sequence of selected genes used for quantitative expression analysis of in-vitro-aged common carp (*Cyprinus carpio*) oocytes and embryos at 5 and 24 HPF

Gene	Forward primer (5'–3')	Reverse primer (5'–3')	Genbank accession no
*fas*	TCGTATTTCTGTGCTGTGCG	TTTCCCTCGTGCTGGTAAGT	XM_042742235.1
*bcl2b*	GGGGCAGAATCATCGCTTTT	ATACTCGGTCATCCAGCCTG	XM_019067767.2
*bax*	CTTCATGAAAGTGGCCCGAG	GCCGACACGCAAAGTAGAAA	KJ174685.1
*tp53*	TGTCCCCACCATGAGAGAAC	TCCCTGTAAAGAGCAAGCGA	XM_042723802.1
*cathepsin D*	TGGACACTGGAACGTCTCTG	TCCCTGAATCAGAGGGATTG	XM_019118951.2
*caspase 8*	GGGGAAGACAACCTGGATGA	CGTGCCCTGTACTCGTCTAT	XM_042725579.1
*caspase 9*	AGAGGGAGTCAGGCTTTTCC	TCCACACTGAAGGAGCTCTG	XM_019066459.1
*caspase 3a*	AGAACTGGATCCTGGTGTGG	AACCTGGTGCCGTAGAGTAC	XM_019110173.2
*Caspase 7*	CAGATCGCGATGCTGAAGAG	TGCAGGTCTGGTCATGGTAG	XM_042735723.1
*gapdh*	GGTGGTGCCAAGAGAGTCAT	AGGAGGCATTGCTGACAACT	XM_042740752.1

### Protein isolation and western blot analysis

A total of 20 mg of oocytes or 20 embryos were lysed in RIPA buffer with a complete protease inhibitor cocktail (Roche, Germany), homogenized on ice with a KIMBLE PELLET PESTLE Cordless Motor (DWK Life Sciences, USA), and centrifuged for 15 min at 10,000 rpm. Total protein concentration was quantified using a Pierce Protein BCA assay kit (Thermo Fisher Scientific, USA). Lysates were denatured in Laemmli buffer (Bio-Rad, USA) with 2-mercaptoethanol (1:10) for 5 min at 95 °C, and 50-µg whole protein lysate were loaded onto a 12% SDS-PAGE gel and transferred to a nitrocellulose membrane. After blocking with 5% skim milk/TBS-T for 1 hour at room temperature (RT), membranes were incubated with primary antibodies anti-caspase 3 (1:500, Abcam, UK) and anti-α-tubulin (1:1,000, Abcam, UK) at 4 °C overnight in 5% BSA/TBS-T. α-Tubulin was used as a protein loading control for western blot analysis. Following TBS-T washing, blots were incubated with an anti-rabbit IgG-HRP secondary antibody (1:1,000, Abcam, UK) for 1 hour at RT. Blots were developed using an ECL kit (BioRad), visualized on a Chemidoc XRS system (BioRad), and quantified using ImageJ (Schneider et al., 2012).

### Caspase-3 fluorometric assay

Caspase 3 is abundantly present in early embryonic development; thus, the active form of caspase 3 was examined along with western blot to confirm the activation of apoptosis (Krumschnabel and Podrabsky, 2009). Caspase 3 enzyme activity was assessed using the EnzChek Caspase-3 Activity Assay Kit (Thermo Fisher Scientific, USA) according to the manufacturer’s instructions. Briefly, 20 mg of oocytes or 20 embryos were lysed in 1× lysis buffer and centrifuged at 13,000 rpm for 20 min, with the supernatant used to determine total protein concentration by BCA assay, and 50 µL of protein lysate was mixed with 50 µL of substrate working solution in a black microtiter plate and incubated for 60 min at RT covered with aluminum foil. Similarly, 50 µL of 1× cell lysis buffer was incubated with the substrate solution as a negative control. Fluorescence was measured at 485-nm excitation and 535-nm emission on a Mithras LB 940 (Berthold Technologies, Germany). Enzyme activity is presented as nM substrate released per mg of protein.

### Statistical analysis

Statistical analyses and graph generation were performed using Prism (9.4.1, GraphPad Software, USA). All the data were normally distributed by the Shapiro–Wilk test. Differences in egg development indices, relative gene expression, relative caspase 3 protein abundance, and caspase 3 enzyme activity from aged oocytes, 5 HPF, and 24 HPF embryos were evaluated by one-way ANOVA followed by Tukey’s multiple comparisons test for post hoc comparisons. Statistical significance was considered at *P ≤ 0.05, **P ≤ 0.01, ***P ≤ 0.001, and ****P ≤ 0.0001.

## Results

### Egg developmental indices decline during in vitro aging

**Figure 2.**
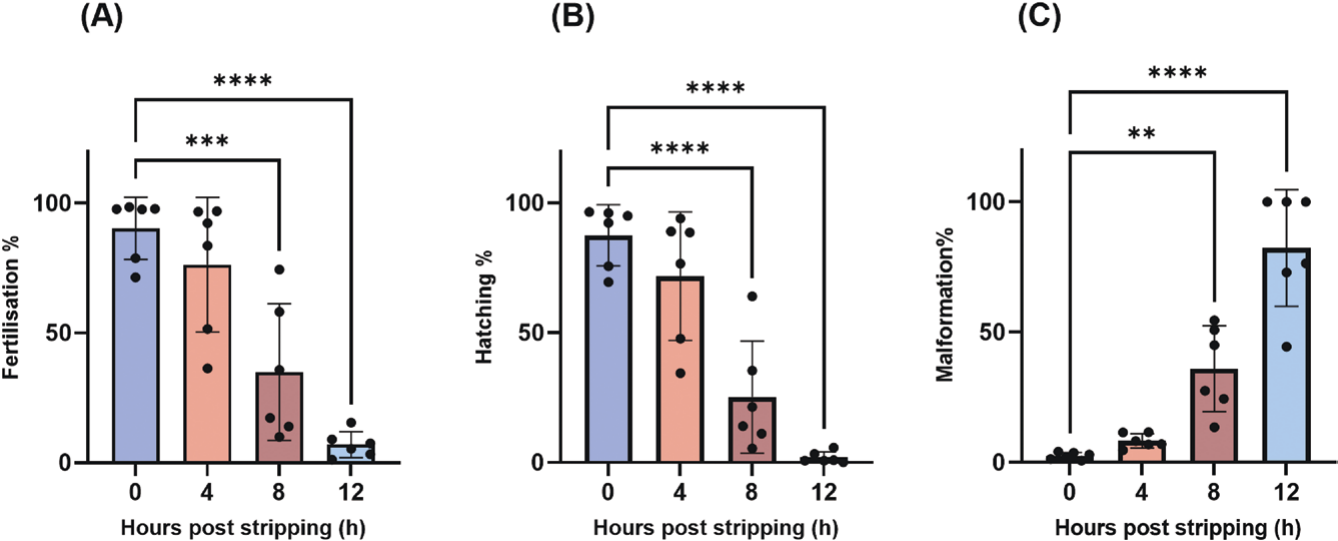
Evaluation of fertilization (A), hatching (B), and malformation rates (C) during in vitro oocyte aging in common carp (*Cyprinus carpio*). Data represent mean ± SD. Statistical significance calculated by one-way ANOVA followed by Tukeys multiple comparison test are shown by *: *P* ≤ 0.05, **: *P* < 0.01, ***: *P* < 0.001, and ****: *P* < 0.0001.

In vitro oocyte aging for up to 4 h had no major effect on fertilization, hatching, or malformation rates (*P* > 0.05) (Figure 2). However, at 8 HPS, fertilization and hatching rates significantly decreased from 90% and 87% to 35% and 25%, respectively (*P* < 0.001) (Figure 2A and B). This decline further continued, reaching 7% and 2% at 12 HPS (*P* < 0.001). The malformation rate increased from 1% at 0 HPS to 36% at 8 HPS and to 82% at 12 HPS (*P* < 0.01) (Figure 2C). Notably, embryos from three individual females showed 100% malformation at 12 HPS (Figure 2C).

### Apoptosis-related gene expression in in-vitro-aged oocytes

**Figure 3.**
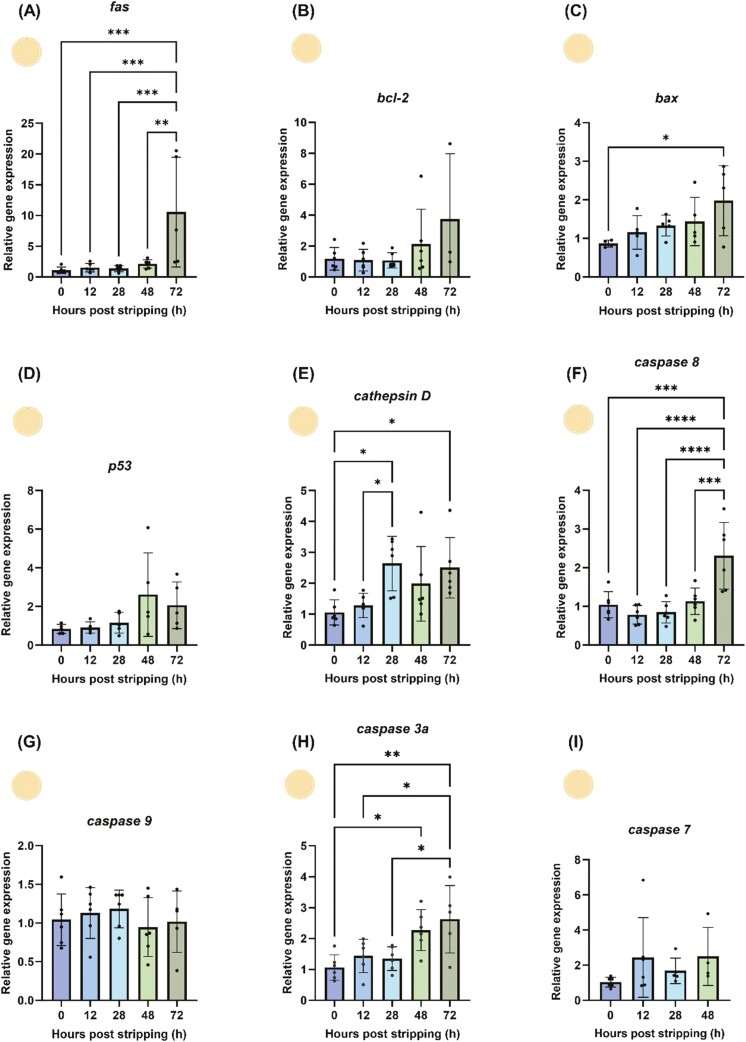
Abundance of apoptosis-related mRNA transcripts during in vitro oocyte aging in common carp (*Cyprinus carpio*). Depicted are the pro- and anti-apoptotic genes *fas* (A), *bcl2* (B), *bax* (C), *tp53* (D), *cathepsin D* (E), *caspase 8* (F), *caspase 9* (G), *caspase 3a* (H), and *caspase 7* (I). Data represent mean ± SD. Statistical significance calculated by one-way ANOVA followed by Tukeys multiple comparison test is shown by *: *P* ≤ 0.05, **: *P* < 0.01, ***: *P* < 0.001, and ****: *P* < 0.0001. Source icons from biorender.

Among the nine candidate genes for apoptosis, five showed significant changes in mRNA abundance between fresh and aged oocytes (Figure 3). The data from 4 and 8 HPS are not shown here as there was no difference among 0, 4, and 8 HPS (*P* > 0.05) (Supplementary Figure 1). The pro-apoptotic gene *fas* exhibited a strong increase at 72 HPS (*P* < 0.001) (Figure 3A). While the anti-apoptotic *bcl-2b* and the pro-apoptotic *p53* did not show relevant regulation during oocyte aging (*P* > 0.05) (Figure 3B and D), the pro-apoptotic *bax* was significantly elevated at 72 HPS (*P* < 0.05) (Figure 3C). *Cathepsin D* expression increased at 28 and 72 HPS (*P* < 0.05), though 48 HPS showed high variance (Figure 3E). The initiator *caspase 8* increased at 72 HPS (*P* < 0.05), whereas *caspase 9* did not change (*P* > 0.05) (Figure 3F and G). Finally, the key player *caspase 3a*, a downstream component of the apoptotic pathway, was strongly upregulated at 48 and 72 HPS (*P* < 0.05) during in vitro oocyte aging (Figure 3H). No significant change was observed with *caspase 7* during in vitro oocyte aging.

### Caspase protein expression and enzyme activity in in-vitro-aged oocytes

**Figure 4.**
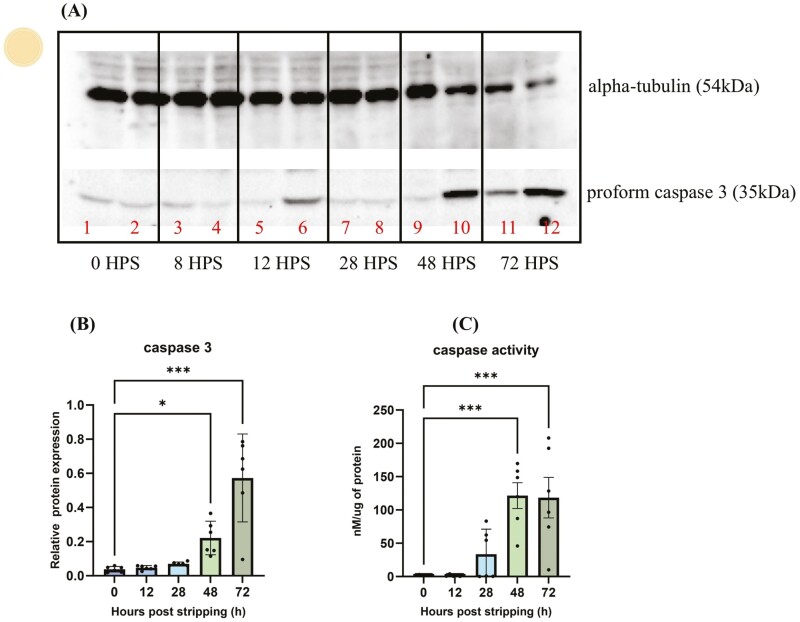
The regulation of zymogenic (A) and active forms of caspase 3 (B) during in vitro aging of oocytes in common carp (*Cyprinus carpio*). Western blot images of caspase 3a with signal at 35 kDa and αtubulin at 54kDa as control from 0, 8, 12, 28, 48, and 72 HPS (C). Data represent mean ± SD. Statistical significance calculated by one-way ANOVA followed by Tukeys multiple comparison test is shown by *: *P* ≤ 0.05, **: *P* < 0.01, ***: *P* < 0.001. Source icons from biorender.

Caspases are present in a zymogenic form and are converted to their catalytically active form during apoptosis. The caspase activity of 4 and 8 HPS was the same as 0 HPS, and thus, data are not shown here (Supplementary Figure 2). In western blotting, the pro-form of caspase 3 significantly increased at 48 and 72 HPS (*P* < 0.05) (Figure 4A and B). Similarly, caspase enzyme activity was higher at 48 and 72 HPS (*P* < 0.001) (Figure 4C).

### Apoptosis-related gene expression in 5 HPF (early blastula) embryos

**Figure 5.**
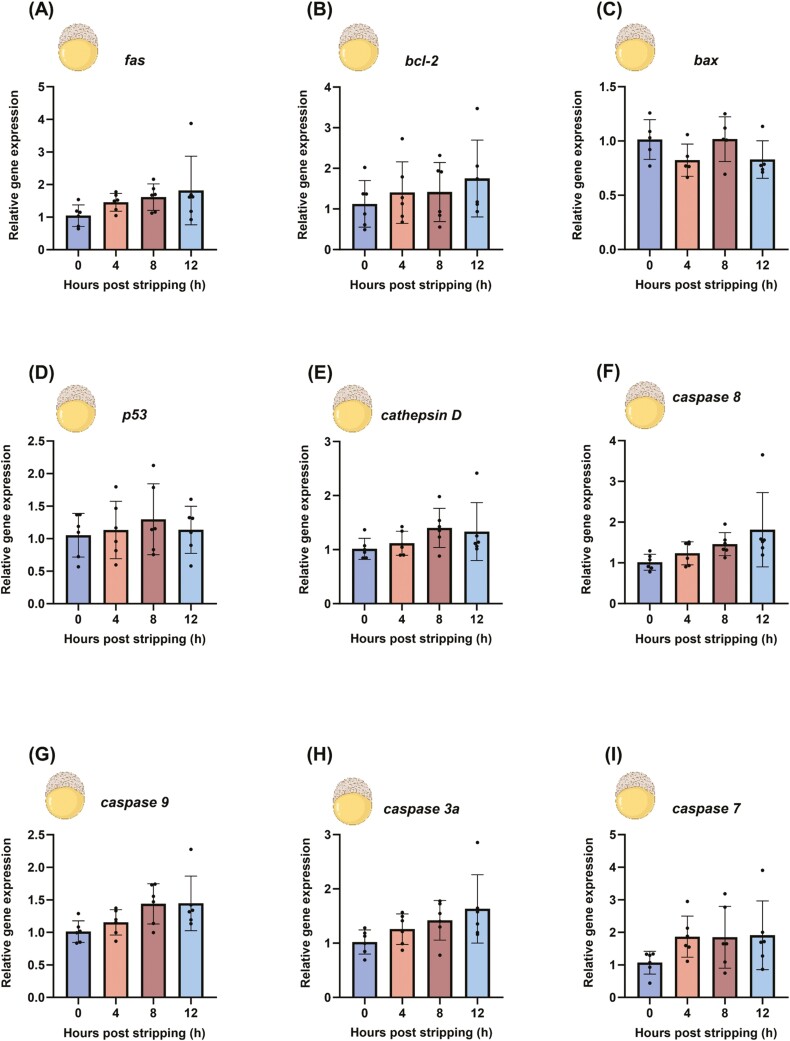
Regulation of pro- and anti-apoptotic genes in 5 HPF (early blastula) embryos from fresh and aged oocytes of common carp (*Cyprinus carpio*). Depicted are the pro- and anti-apoptotic genes *fas* (A), *bcl2* (B), *bax* (C), *tp53* (D), *cathepsin D* (E), *caspase 8* (F), *caspase 9* (G), *caspase 3a* (H), and *caspase 7* (I). Data represent mean ± SD. Statistical significance calculated by one-way ANOVA followed by Tukeys multiple comparison test is shown by *: *P* ≤ 0.05. Source icons from biorender.

No significant changes were observed in apoptosis-related gene expression in the 5 HPF embryos regardless of the oocyte aging time-point (0, 4, 8, and 12 HPS) (*P* > 0.05) (Figure 5). This indicates that apoptosis-related genes do not play a relevant role at this early stage of development.

### Caspase enzyme activity in 5 HPF (early blastula) embryos

**Figure 6.**
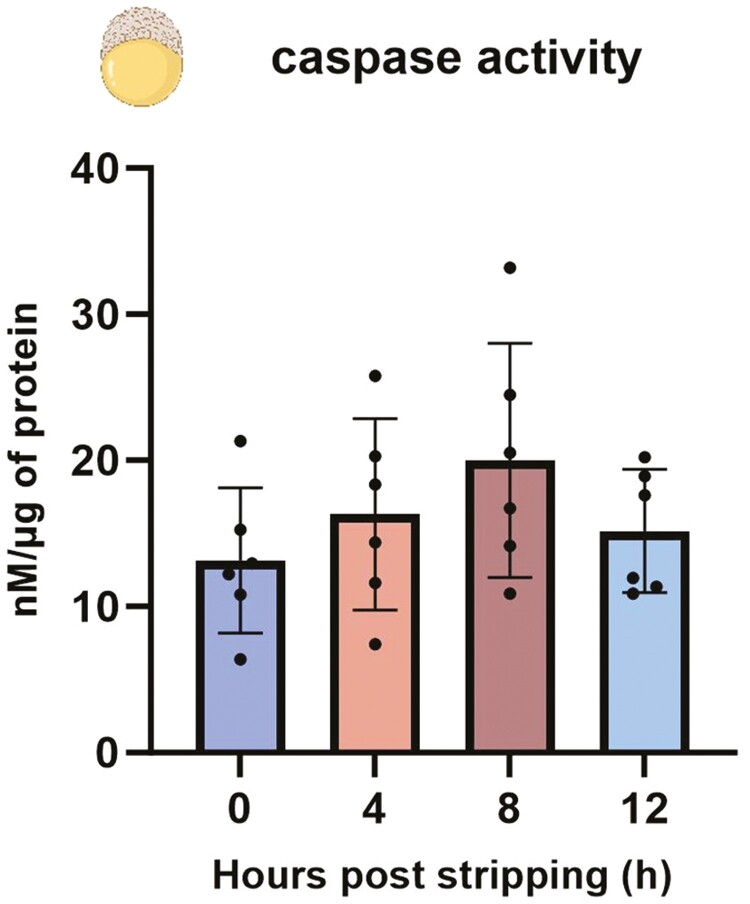
The caspase 3 enzyme activity in 5 HPF (early blastula) embryos from fresh and aged common carp (*Cyprinus carpio*) oocytes. Data represent mean ± SD. Statistical significance calculated by one-way ANOVA followed by Tukeys multiple comparison test is shown by *: *P* ≤ 0.05. Source icons from biorender.

Consistent with gene expression data, no changes in enzyme activity were observed in 5 HPF embryos originating from fresh and aged oocytes (*P* > 0.05) (Figure 6).

### Apoptosis-related gene expression in 24 HPF (bud stage) embryos

**Figure 7.**
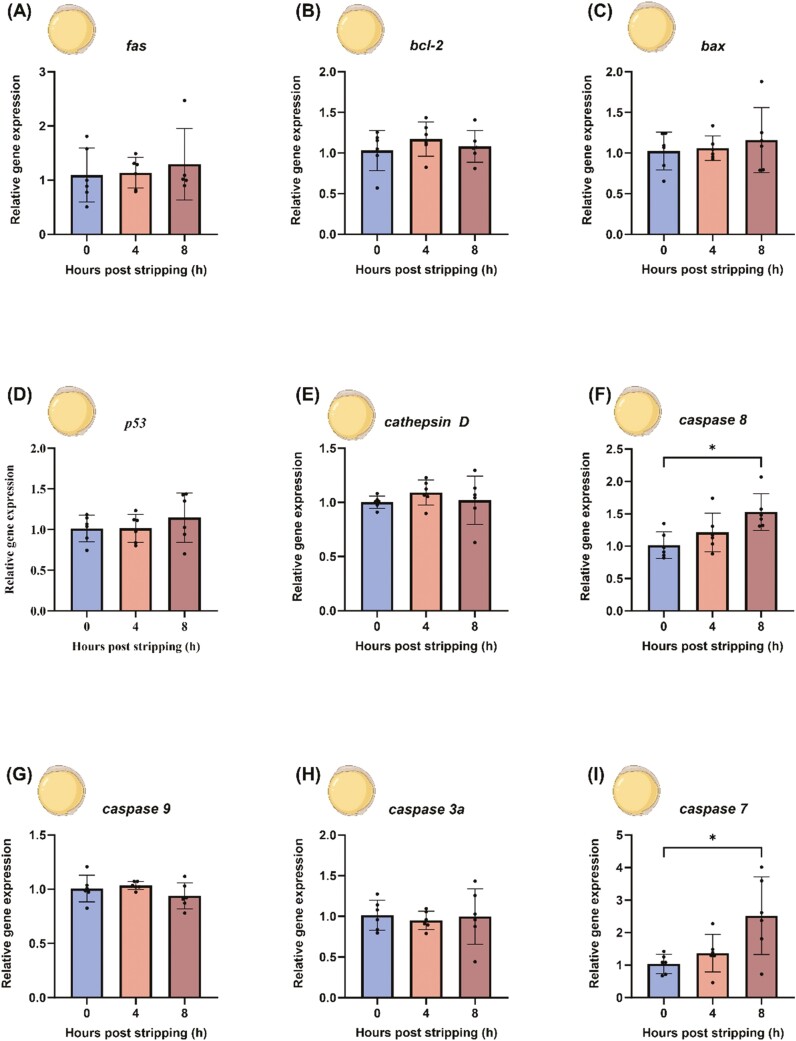
Regulation of pro- and anti-apoptotic genes in 24HPF embryos from fresh and aged common carp (*Cyprinus carpio*) oocytes. Depicted are the pro- and anti-apoptotic genes *fas* (A), *bcl2* (B), *bax* (C), *tp53* (D), *cathepsin D* (E), *caspase 8* (F), *caspase 9* (G), *caspase 3a* (H), and *caspase 7* (I). Data represent mean ± SD. Statistical significance calculated by one-way ANOVA followed by Tukeys multiple comparison test is shown by *: *P* ≤ 0.05. Source icons from biorender.

In 24 HPF embryos, apoptotic genes such as *fas*, *bcl2b*, *bax*, *tp53*, and *cathepsin D* showed constant expression levels in embryos originating from 0-, 4-, and 8-h-aged oocytes (*P* > 0.05) (Figure 7A–E). Due to the low fertilization rate from 12-h-aged oocytes, sample numbers were limited, and thus, there is no relative expression and western blot data of 24 HPF embryos from 12-h-aged oocytes. *Caspase 9* and *caspase 3a* did not exhibit significant differential expression between embryos from fresh and aged oocytes (*P* > 0.05) (Figure 7G and H). In contrast, the initiator *caspase 8* and the effector *caspase 7* showed a significant increase in embryos from 8-h-aged oocytes compared with embryos from fresh oocytes (*P* < 0.05) (Figure 7F and I).

### Caspase protein expression and enzyme activity in 24 HPF (bud stage) embryos

**Figure 8.**
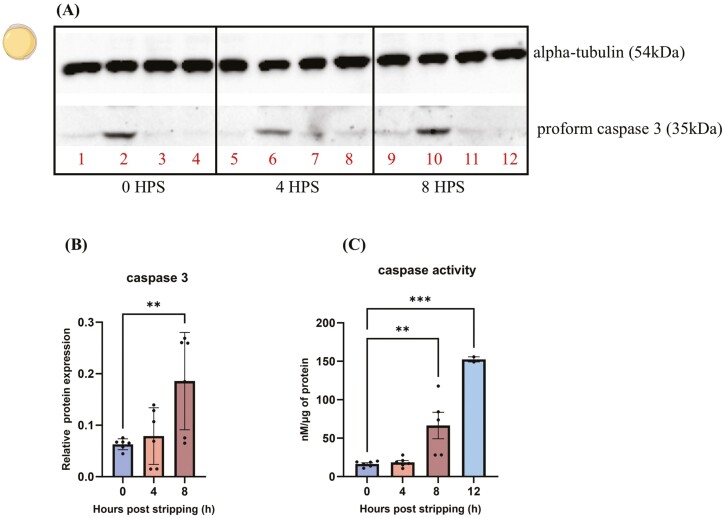
The regulation of zymogenic (A) and active forms of caspase 3 (B) in 24 HPF embryos from fresh and aged common carp (*Cyprinus carpio*) oocytes. Western blot images of caspase 3a with signal at 35 kDa and αtubulin at 54 kDa as control in embryos from 0, 4, and 8 HPS (c). Data represent mean ± SD. Statistical significance calculated by one-way ANOVA followed by Tukeys multiple comparison test is shown by *: *P* ≤ 0.05, **: *P* < 0.01, ***: *P* < 0.001. Source icons from biorender.

The pro-form of caspase 3 significantly increased in embryos originating from 8-h-aged oocytes (*P* < 0.01) (Figure 8A and B). Caspase enzyme activity significantly increased in embryos fertilized from 8- and 12-h-aged oocytes (*P* < 0.001) (Figure 8C).

## Discussion

Fertilization rate is a good estimator of gamete quality; however, it is technically unfeasible to assess in all fish species (Bobe and Labbé, 2010). Delayed cleavage in embryos originating from aged oocytes (Azuma et al., 2003) is a limitation in calculating the fertilization rate in aged oocytes. Therefore, hatching and malformation rates were also estimated to understand the developmental potential of aged oocytes. The normal developmental competence of common carp oocytes decreased with in vitro aging, similar to previous reports with other fish species (Flajšhans et al., 2007; Samarin et al., 2011; Nomura et al., 2013; Waghmare et al., 2021a). The present study results imply that common carp oocytes can be stored for 4 h with minimal loss of competency even without any external storage medium. However, the duration for maintaining fertility postovulation varies with species and storage conditions (Samarin et al., 2015b). For instance, short-term storage of *Prochilodus marggravii* oocytes showed no structural changes up to 2-h postspawning but reduced fertilization capacity and resulted in deformed larvae (Rizzo et al., 2003). Complete loss of egg fertility was observed without any consequences on transcripts related to oxidative stress, mitochondrial dysfunction, and apoptosis in 8-h-aged African catfish (*Clarias gariepinus*) oocytes (Samarin et al., 2018). In vivo aging of common carp oocytes negatively affected the egg developmental competence without affecting the oxidation status (Samarin et al., 2019a). The global and specific histone modifications remained the same, though fertilization capacity declined in 12-h-aged common carp oocytes (Waghmare et al., 2021b). Thus, the mechanism behind the loss of developmental competence due to postovulatory oocyte aging remains elusive.

The association of FasL with the Fas receptor forms the death-inducing signaling complex, which activates caspase 8 (Algeciras et al., 2002). Activated caspase 8 promotes the release of cytochrome c by activating Bid (Fouqué et al., 2014) or directly activates caspase 3 to execute apoptosis (Elmore, 2007). Our study suggests that the death of in-vitro-aged carp oocytes involves the Fas/FasL system, as indicated by the increased *fas* mRNA level at 72 HPS. This finding is consistent with the increase in Fas in fragmented sea bream (*Sparus aurata*) oocytes (Carnevali et al., 2003) and in-vitro-aged mice oocytes (Zhu et al., 2016). The ratio of proapoptotic and antiapoptotic Bcl-2 family proteins determines cell fate. An excess of bcl2 results in cell survival, while an excess of bax leads to cell death (Adams and Cory, 1998). Bcl2 is a transmembrane protein that maintains mitochondrial membrane potential and calcium homeostasis, ensuring cell survival (Cory and Adams, 2002). Bax disrupts the mitochondrial membrane, promoting the release of cytochrome c, which activates caspases (Oltvai et al., 1993). In our study, the mRNA level of *bcl2b* remained unchanged during in vitro aging for 72 h, consistent with in-vivo-aged common carp oocytes (Samarin et al., 2019a). However, *bax* showed significant increases at the very advanced stage of aging, similar to the upregulation observed in in-vivo-aged common carp oocytes (Samarin et al., 2019a). The constant level of *bcl2b* and the increase in *bax* suggest that apoptosis in aged common carp oocytes is regulated by fas-mediated bax activation without altering bcl2b.

Cathepsins are lysosomal proteases that disrupt mitochondrial membranes and activate apoptosis (Paquet et al., 2005). A significant increase in *cathepsin D* was observed in 28- and 72-h-aged oocytes, consistent with results in zebrafish oocytes (Konar et al., 2024) and sea bream oocytes exhibiting apoptosis (Carnevali et al., 2003). In contrast, rainbow trout (*Oncorhynchus mykiss*) (Aegerter et al., 2005) and African catfish aged oocytes exhibited no change in the mRNA level of *cathepsin D* (Samarin et al., 2018). Oocyte cell death might be signaled by different cathepsins in rainbow trout and African catfish since a high level of *cathepsin Z* was found in aged rainbow trout oocytes (Aegerter et al., 2005). The alternative mechanism of cathepsins activating apoptosis is through direct activation of caspases (Conus et al., 2008). Activation of caspases is the irreversible step in the apoptotic pathway and most morphological features of apoptosis are due to caspase activation (reviewed by Elmore, 2007). In our study, the mRNA abundance of *caspase 3a* and *caspase 8* significantly increased. The zymogenic and active forms of caspase 3 also increased with aging time, which depicts the activation of apoptosis as the endpoint of oocyte aging. This is consistent with the time-dependent increase in caspase 3 enzyme activity observed in unfertilized *Xenopus* eggs after spawning (Tokmakov et al., 2011). Thus, the apoptosis is initiated after 48 h of aging in common carp based on the results obtained in this study.

Matured oocytes are transcriptionally silent until the initiation of zygotic transcription and rely on stored mRNAs for early embryo development (Lubzens et al., 2017). Posttranslational histone modifications are one of the crucial mechanisms behind maternal zygotic transition. Hyperacetylation of histones, an indicator of active transcription, was observed in common carp oocytes aged 28 h (Waghmare et al., 2021b). Spontaneous activation is a prerequisite for apoptosis activation, occurring after complete loss of fertilization in advanced stages of oocyte aging (Tokmakov et al., 2011). Thus, the increase in *fas*, *bax*, *cathepsin D*, and *caspase 8* and *3a* transcripts in our study could be linked to spontaneous activation of oocytes, ceasing transcriptional silence and presumably triggering cell death.

Apoptosis is a homeostasis mechanism during embryonic development, removing unwanted and damaged cells (Krumschnabel and Podrabsky, 2009). Apoptotic genes such as *bcl2*, *mcl1*, *caspase 8*, *9*, and *3* are maternally transferred to embryos (Lanes et al., 2012). Our results are the first to study apoptosis-related genes in embryos from aged common carp oocytes. In our study, there was no differential expression of apoptosis-related genes in 5 HPF embryos from fresh and aged oocytes, likely due to their dependence on maternally deposited apoptotic transcripts. The apoptosis machinery in early blastula embryos is inactive until the mid-gastrula stage (Hensey and Gautier, 1997; Ikegami et al., 1997; Mizoguchi et al., 1999). Thus, oocyte aging has no effect on apoptosis surveillance in 5 HPF embryos.

The initiator *caspase 8* and effector *caspase 7* were upregulated in 24 HPF (bud stage) embryos from aged oocytes. Similarly, overexpressed caspase 8 in zebrafish embryos led to apoptotic cell death (Eimon et al., 2006). In our study, the zymogenic and active forms of caspase 3 were upregulated in 24 HPF embryos from aged oocytes, indicating intensified apoptosis. This may contribute to malformations, as damage-induced apoptosis is responsible for structural malformations during early embryo development (Sawant et al., 2014; Gutiérrez-Noya et al., 2021). The undeveloped fertilized embryos from 12-h-aged oocytes might be died by apoptosis as indicated by high amount of active caspase 3 and resulting in high mortality rate at 24-h postfertilization. Future studies should include histological analysis of embryos derived from aged oocytes to gain a deeper understanding of developmental apoptosis.

## Conclusion

Decline in fertilization and hatching rates, accompanied by an increase in malformation rates, was observed as the effects of in vitro aging in common carp oocytes. The upregulation of apoptosis-related genes and the activation of caspase 3 highlight the significant role of apoptosis in the degradation of oocyte quality over time. The findings of this study suggest that the Fas/FasL system and increase in proapoptotic genes (*bax*, *cathepsin D*, *caspase 8*, *caspase 9*, and *caspase 3a*) are critical in this process. Notably, intensified apoptosis in 24 HPF embryos derived from aged oocytes underscores the potential of oocyte aging for long-term developmental consequences. Future studies should focus on histological analyses to understand the consequences of aging on structural apoptosis regulation. Further exploration of posttranscriptional regulation will deepen our understanding of the spontaneous activation underlying oocyte aging. This study is the first to confirm the activation of apoptosis in aged common carp oocytes, which is important to understand the fate of unfertilized oocytes. This research not only enhances our understanding of oocyte aging in common carp but also provides valuable insights that could inform gamete preservation and fertility management strategies in other fish species.

## Supplementary Material

skaf002_suppl_Supplementary_Figures_S1-S4

## Data Availability

The raw data supporting the conclusions of this article will be made available by the authors without undue reservation.

## Abbreviations

PGC: primordial germ cell
HPS: hours post stripping
HPF: hours post fertilization
CPH: carp pituitary homogenate
