# Supplementary material for: Aging oocytes: exploring apoptosis and its impact on embryonic development in common carp (Cyprinus carpio)
Source: J Anim Sci. 2025 Jan 6;103:skaf002. doi: 10.1093/jas/skaf002 (PMC11757700; doi:10.1093/jas/skaf002)
Supplement: skaf002_suppl_Supplementary_Figures_S1-S4 [file skaf002_suppl_supplementary_figures_s1-s4.docx]

Supplementary Figure 1
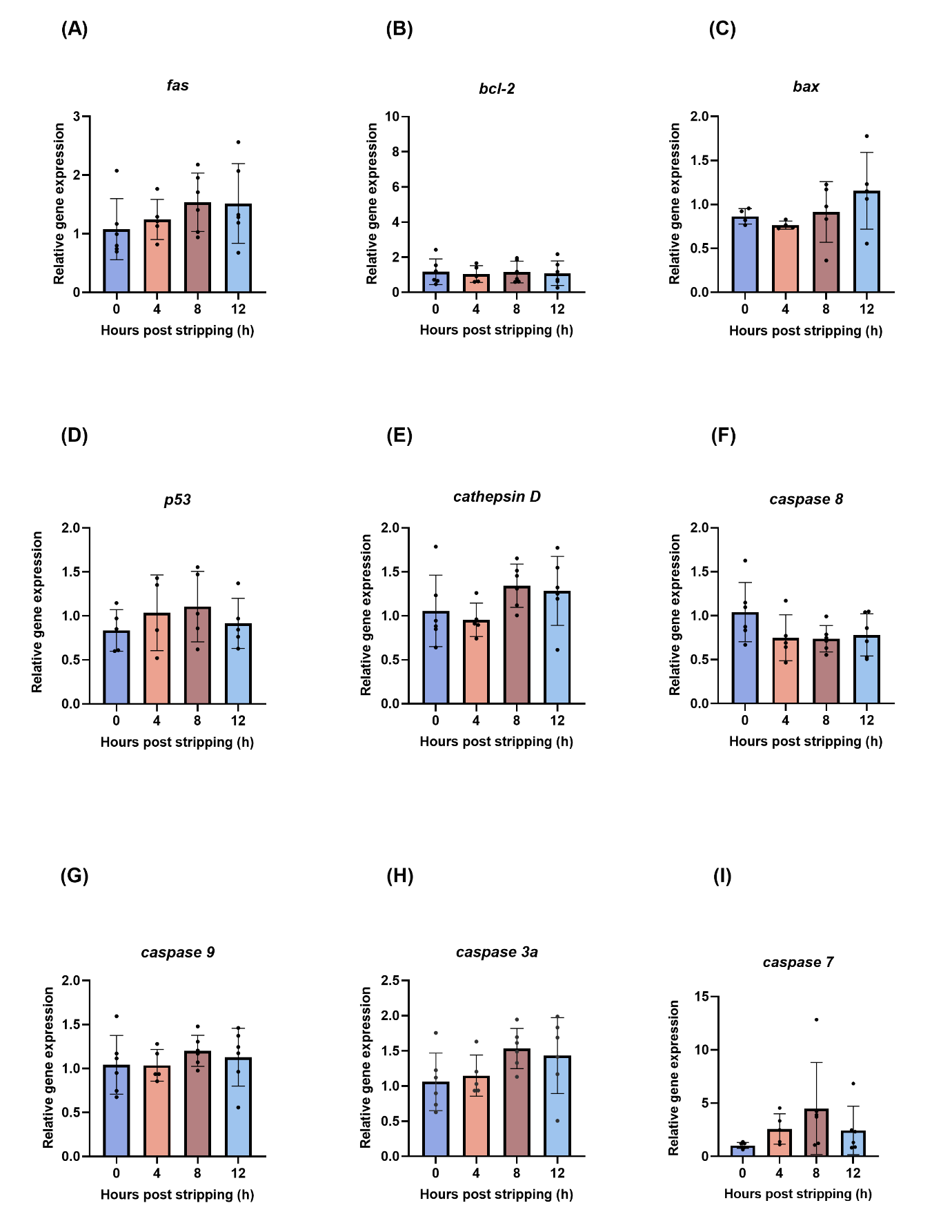


Figure 3. Abundance of apoptosis-related mRNA transcripts in *in vitro* aged *Cyprinus carpio* oocytes. Depicted are the pro- and anti-apoptotic genes fas (A), bcl2 (B), bax (C), tp53 (D), cathepsin D (E), caspase 8 (F), caspase 9 (G), caspase 3a (H), caspase 7 (I). Data represents mean ± SD. Statistical significance calculated by one-way ANOVA followed by tukeys multiple comparison test is shown by *: p ≤ 0.05.


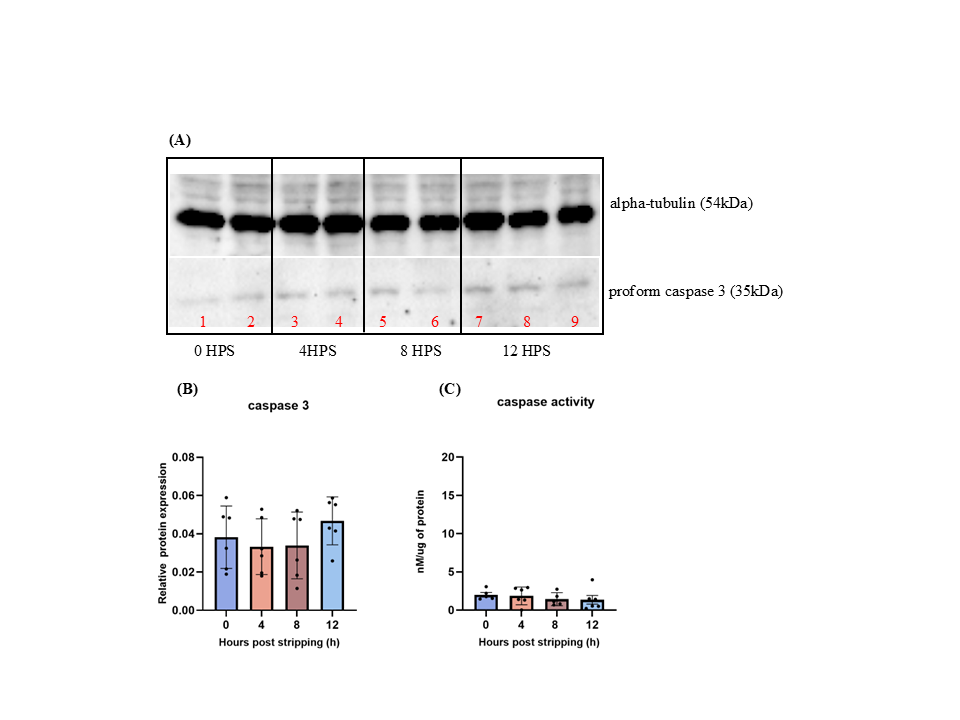
Figure 2

Figure 4. The regulation of zymogenic (A) and active forms of caspase 3 (B) during in vitro aging of *Cyprinus carpio* oocytes. Western blot images of caspase 3a with signal at 35kDa and αtubulin at 54kDa as control from 0,4,8, and12 HPS (C). Data represents mean ± SD. Statistical significance calculated by one-way ANOVA followed by tukeys multiple comparison test is shown by *: p ≤ 0.05.
